# Prevalence and determinants of double and triple burden of malnutrition among mother–child pairs in Malawi: a mapping and multilevel modelling study

**DOI:** 10.1017/S1368980024002064

**Published:** 2024-10-21

**Authors:** Jessie Jane Khaki, Peter M Macharia, Lenka Beňová, Emanuele Giorgi, Aline Semaan

**Affiliations:** 1 Centre for Health Informatics, Statistics and Computing (CHICAS), Lancaster University, Lancaster, UK; 2 Malawi Liverpool Wellcome (MLW) Programme, Blantyre, Malawi; 3 School of Global and Public Health, Kamuzu University of Health Sciences, Blantyre, Malawi; 4 Department of Public Health, Institute of Tropical Medicine, Antwerp, Belgium; 5 Population & Health Impact Surveillance Group, Kenya Medical Research Institute-Wellcome Trust Research programme, Nairobi, Kenya

**Keywords:** Double burden, Triple burden, Malnutrition, Malawi, Mother–child pairs

## Abstract

**Objective:**

To establish the prevalence of double burden of malnutrition (DBM) and triple burden of malnutrition (TBM) among mother–child pairs in Malawi and explore their geographical distribution and associated multilevel factors.

**Design:**

Cross-sectional study using secondary data from the 2015–2016 Malawi Demographic and Health Survey using a mixed effects binomial model to identify multilevel factors associated with DBM and TBM. Georeferenced covariates were used to map the predicted prevalence of DBM and TBM.

**Setting:**

All twenty-eight districts in Malawi.

**Participants:**

Mother–child pairs with mothers aged 15–49 years and children aged below 60 months (*n* 4618 pairs) for DBM and between 6 and 59 months (*n* 4209 pairs) for TBM.

**Results:**

Approximately 5·5 % (95% confidence interval (CI): 4·7 %, 6·4 %) of mother–child pairs had DBM, and 3·1 % (95 % CI: 2·5 %, 4·0 %) had TBM. The subnational-level prevalence of DBM and TBM was highest in cities. The adjusted odds of DBM were threefold higher (adjusted Odds Ratio, AOR: 2·8, 95 % CI: 1·1, 7·3) with a higher proportion of wealthy households in a community. The adjusted odds of TBM were 60 % lower (AOR: 0·4; 95 % CI: 0·2, 0·8) among pairs where the women had some education compared with women with no education.

**Conclusions:**

Although the prevalence of DBM and TBM is currently low in Malawi, it is more prevalent in pairs with women with no education and in relatively wealthier communities. Targeted interventions should address both maternal overnutrition and child undernutrition in cities and these demographics.

Malnutrition, including micronutrient deficiencies, excesses or imbalances, causes serious and long-lasting adverse outcomes at both individual and community levels. Nearly half (45 %) of all deaths among under-five children globally are due to nutrition-related factors, and the burden is higher in low- and middle-income countries^(1)^. Since 2000, there has been substantial progress in reducing the burden of undernutrition among under-five children^(2)^. The global prevalence of stunting, which is the most common type of child malnutrition, has reduced from 32·6 % in 2000 to 22·2 % in 2017^(2)^. However, a recent World Health Organization (WHO) report cited that populations in 88 % of 141 countries experienced multiple types of malnutrition, including child stunting and overweight in women^(2)^. The report further highlighted that forty-one (29 %) of the countries, thirty of which are in Africa, experience a high burden of child stunting (≥ 20 %) and overweight and obesity in adult women (≥ 35 %).

The coexistence of undernutrition (such as stunting, wasting or micronutrient deficiency) and overnutrition (obesity or overweight) is defined as double burden of malnutrition (DBM)^(3)^. The WHO states that DBM can occur at the individual level (e.g. coexistence of overnutrition with mineral or vitamin deficiencies in one individual), household level (e.g. nutritional anaemia in a child and overnutrition in another member of the household) and population level (e.g. the existence of a burden of undernutrition and overnutrition in the same community such as a village, district or country)^(3)^. DBM among mother–child pairs is defined as the coexistence of undernutrition (wasting, stunting or underweight) in the child and overnutrition (overweight or obesity) in the mother^(4)^. Furthermore, a mother–child pair can also have a child with overnutrition and undernutrition in the mother. The triple burden of malnutrition (TBM) refers to the coexistence of micronutrient deficiencies and undernutrition in children and maternal overnutrition^(4)^. Similarly, TBM among mother–child pairs can include overnutrition in the child and the coexistence of undernutrition and micronutrient deficiencies in the mother. Childhood malnutrition is associated with multiple adverse outcomes such as delayed cognitive development and mortality^(5)^. Likewise, overnutrition in adults is associated with an increased risk of acquiring non-communicable diseases such as high blood pressure and diabetes and poor pregnancy outcomes among women^(6)^. DBM and TBM are, therefore, increasingly being recognised as public health threats because of the risks they pose to both the mother and child and the underlying complexity resulting in the coexistence of different types of malnutrition.

The burden of DBM and TBM varies between countries. Although the global mother–child pair prevalence of DBM and TBM is unknown, the prevalence of household-level DBM ranges between 3 % and 35 % across 126 low- and middle-income countries, with the highest prevalence reported in Southern Africa, South America and Asia^(7,8)^. For Southern Africa, a study carried out in 2021 in twenty-three countries of the region estimated the prevalence of household-level DBM and TBM to be 8 % and 5 %, respectively^(9)^.

Malawi has been experiencing DBM. The most recent 2015–2016 Malawi Demographic and Health Survey (MDHS) reported that the prevalence of stunting (37 %), underweight (12 %), overweight (5 %) and wasting (3 %) among under-five children declined compared with the 2010 MDHS (stunting = 47 %, underweight = 13 %, overweight =8 % and wasting = 4 %)^(10,11)^. However, the prevalence of overweight among women of reproductive age increased from 17 % in 2010 to 21 % in 2015–2016. Further, the prevalence of anaemia among under-five children decreased from 73 % in 2004 to 63 % in 2010 and remained at that level in 2015–2016, suggesting a stall in the reduction. Although there has been extensive research in Malawi on factors associated with various forms of malnutrition among children and overnutrition among women of reproductive age, research looking at the co-occurrence of undernutrition, micronutrient deficiencies among children and overnutrition among their mothers in Malawi is scarce^(12–14)^.

There is limited research on geographical disparities in DBM and TBM among mother–child pairs in low- and middle-income countries. Tarekegn *et al.* used the Anselin Local Moran’s *I* test to identify hotspots for DBM and TBM among mother–child pairs in Ethiopia^(15)^. In Kenya, Kasomo *et al.* used a Bayesian geoadditive regression model to identify factors associated with DBM among women and to identify areas with a high burden of DBM^(16)^. Both studies showed that the distribution of DBM and TBM varies across space. In addition to the spatial studies, other previous research used multilevel analyses to determine factors associated with TBM^(17)^.

The objectives of the present study were to (1) estimate the prevalence of DBM and TBM among mother–child pairs, (2) examine the variability in the prevalence of DBM and TBM geographically and (3) identify individual, household and community-level factors associated with DBM and TBM among mother–child pairs, in Malawi.

## Methods

### Data

The most recent 2015–2016 MDHS data were used for this study^(10)^. The Demographic and Health Surveys (DHS) are nationally representative cross-sectional household surveys that are carried out in low- and middle-income countries for tracking health and demographic indicators^(18)^. Respondents for the 2015–2016 MDHS were sampled using a two-stage process, which was guided by a sampling frame generated from the 2008 Malawi Population and Housing Census^(10)^. Details on the MDHS sampling methodology can be found in other reports^(10)^. In summary, the 2015–2016 MDHS sampled 850 enumeration areas (EA) in all the twenty-eight districts of Malawi in the first sampling stage. In the second stage, thirty-three households in each rural cluster and thirty households in each urban cluster were selected. Enumerators used a global positioning system to identify the central point of each EA and to collect coordinates (latitude and longitude). The DHS programme displaces the coordinates by up to 2 km in urban areas and up to 5 km in 99 % of the rural areas and by 10 km in the remaining 1 % of EA in rural areas for anonymization purposes^(19)^. Coordinate displacements ensure that points remain within the same administrative boundaries of the EA^(19)^.

### Study population

The population of interest in the study was living together in a household mother–child pairs where the child was less than 60 months (5 years) old at the time of the survey. We used the women’s and children’s recode datasets that contain information on women of reproductive age (15–49 years), their most recent birth and their children. Anthropometry and biomarker data were collected from members of a sub-sample of the interviewed households. Height and weight measurements were taken from women aged between 15 and 49 years and children aged between 0 and 59 months in the eligible households^(10)^. To measure hemoglobin (Hb) levels for determining anaemia status, blood specimens were collected from a sub-sample of households eligible for anthropometry data collection.

The analysis sample for DBM included women of reproductive age (15–49 years) with all their under-five children residing in the same household. The analysis of TBM was restricted to a subset of women of reproductive age and their children aged between 6 and 59 months because the DHS collected Hb levels for this child age group only^(10)^. Pregnant women at the time of the survey or who gave birth in the two months before the survey were excluded from the sample to avoid their pregnancy weight biasing their body mass index (BMI)^(10,20)^. We also excluded women and children whose anthropometric measurements were not recorded^(20)^. Furthermore, we excluded children whose dates of birth were missing or unknown and also excluded children with anthropometric measurements outside of plausible ranges as defined by the Guide to DHS Statistics^(20)^. The sample inclusion flow chart is provided in the online supplementary material, Supplemental Information I.

### Outcome and independent variables definition

This study had two outcome variables: double burden of malnutrition (DBM) and triple burden malnutrition (TBM) among mother–child pairs. The operational definitions for DBM and TBM were adapted from previous studies and are presented in Table 1, along with definitions of malnutrition indicators such as stunting and wasting available in the MDHS^(4,15)^.

**Table 1.** Outcome variable definition as adapted from previous DBM and TBM studies^(4,15,21)^

Indicator	Unit of analysis	Definition
Wasting (weight for height Z-score (WHZ))	Children 0–59 months	1 = wasted (WHZ < –2 s d), 0 = not wasted
Stunting (height/length for age Z-score (HAZ))	Children 0–59 months	1 = stunted (HAZ < –2 sd), 0 = not stunted
Underweight (weight for age Z-score (WAZ))	Children 0–59 months	1 = underweight (WAZ < –2 sd), 0 = not underweight
Child undernutrition	Children 0–59 months	1 = wasted, stunted or underweight, 0 = no undernutrition
Child overnutrition	Children 0–59 months	1 = overweight (WAZ > 2 sd), 0 = not overweight
Child anaemia	Children 6–59 months	1 = anaemic (Hb level < 11·0 g/dl), 0 = not anaemic
Maternal overnutrition (overweight or obesity) based on BMI	Women 15–49 years	1 = overweight (BMI 25·0–29·9 weight (kg)/height(m^2^)) or obese (BMI 30/kg/m^2^), 0 = normal/underweight BMI
Maternal underweight	Women 15–49 years	1 = mildly thin (BMI 17·0–18·4 kg/m^2^) or moderately or severely thin (BMI < 17·0 kg/m^2^), 0 = normal/overweight or obese BMI
Maternal short height	Women 15–49 years	1 = height < 145 cm, 0 = normal height
Maternal undernutrition	Women 15–49 years	1 = maternal underweight or short height, 0 = normal
Maternal anaemia	Women 15–49 years	1 = anaemic (Hb level < 12·0 g/dl), 0 = not anaemic
Double burden of malnutrition (DBM)	Women 15–49 years and their children 0–59 months from their most recent birth, including multiple births	1 = mother’s (maternal) overweight or obesity *and* child undernutrition (wasting = 1 or stunting = 1 or underweight = 1) *or* maternal undernutrition *and* child overnutrition, 0 = no DBM
Triple burden of malnutrition (TBM)	Women 15–49 years and their children 6–59 months from their most recent birth, including multiple births	1 = maternal overweight or obesity and undernourished and anaemic child (child anaemia = 1) *or* child overweight *and* maternal undernutrition and maternal anaemia (maternal anaemia = 1), 0 = no TBM

The study explored associations between the outcomes and independent variables at the individual, household and community levels. An initial selection of variables was based on the WHO conceptual framework for the DBM (see online supplementary material, Supplemental Information II) and previous research looking at the prevalence and burden of DBM and TBM^(4,15,21,22)^. These variables are listed in Table S1 in the online supplementary material, Supplemental Information II, along with the variable label. The community-level variables were aggregated from the individual/household level to the EA (i.e. cluster) level. For instance, we generated a new variable and assigned a one to the households in the middle, rich or richest wealth quintiles and a zero to the households in the poor/poorest quintiles. The variable capturing the percentage of households in at least the middle wealth quintile in a cluster was, therefore, computed by taking the sum of the new variable divided by the total number of households sampled in that cluster.

The following gridded raster covariates were used in the mapping analysis: precipitation, nightlights, elevation, temperature, aridity, antenatal visits during pregnancy, female literacy and percentage of children who had received all basic vaccinations. These variables have been shown to be associated with nutrition-related indicators and have been included in previous modelling and mapping studies^(17,23–28)^. The variables were also selected based on the WHO conceptual framework (see online supplementary material, Supplemental Information II) of DBM and conceptual frameworks for mother–child pair DBM from previous research^(29,30)^. Details on the sources of the variables and their spatial and temporal resolution are given in the online supplementary material, Supplemental Information II.

### Statistical analyses

Outcome and independent variables were explored by tabulating frequencies for the categorical variables and computing the mean and standard deviations for the continuous variables. Bivariate analyses were carried out using a Chi-square test for categorical variables and *t*-test for continuous variables. To avoid including multiple independent variables that were highly correlated, we fitted a logistic regression model to the outcome variables and computed the variance inflation factors of the independent variables. All the variables had a variance inflation factor of less than 3 and were included in the multilevel models^(31,32)^.

The multivariable analysis first considered a binomial mixed model (three-level multilevel model where mother–child pairs reside within a household that is in a community/EA) to investigate multilevel risk factors of TBM and DBM. Mixed models are valuable for analysing data with hierarchical or nested structures, allowing for the incorporation of both fixed and random effects^(33)^. The clustering variables that were used in the study were the household number and the EA, which is coded as a cluster number in the DHS data. A three-level multivariable binomial mixed model was fitted to investigate the determinants of DBM and TBM at the individual, household and cluster levels where mother–child pairs (individual, Level 1) are in households (Level 2), which are clustered within an EA (community level, Level 3).

#### Spatial modelling

To map DBM and TBM, we adapted a methodology utilised in estimating and mapping other health-related outcomes that are derived from multiple indicators^(34,35)^. Each of the indicators contributing to DBM and TBM (child stunting, child wasting, child underweight, child overnutrition, child anaemia, maternal undernutrition, maternal short height, maternal anaemia and maternal overnutrition) was individually considered in our approach^(34)^. We examined multicollinearity and selected between closely associated spatial covariates as described above^(32,36)^. We then fitted binomial generalised mixed models to each of the nine indicators. Random effects extracted from the binomial mixed models were used to fit variograms, which confirmed the absence of spatial correlation in all nine indicators, as depicted in Fig. S4 and Fig. S5 in the online supplementary material. We, therefore, mapped DBM and TBM using the non-spatial binomial mixed models as done in other non-spatial mapping studies since spatial dependence is required when using geospatial methods^(36–38)^. We computed the predicted prevalence of each of the nine indicators and combined them to produce the predicted prevalence of DBM and TBM at the pixel level (3 km × 3 km grid) and district level^(34)^. We utilised the multivariate Gaussian approximation of the maximum likelihood estimator to compute confidence intervals (CIs) for the estimates as employed in previous non-spatial mapping studies^(38)^.

We generated new level-weights according to the recent guidance by the DHS methodology team^(39)^. All analyses adjusted for the weights and were carried out at a 5 % significance level using Stata version 15 and R. We further adjusted for the stratification. Statistical details for the multilevel model and mapping analyses are available in the online supplementary material.

## Results

We analysed data for a total of 4618 for DBM and 4209 for TBM (Level 1 data) mother–child pairs. The DBM mother–child sample was from 3661 households (Level 2) within 848 communities (Level 3). The TBM mother–child sample was from 3442 unique households (Level 2) within 844 communities (Level 3). Among the 4618 mother–child pairs in the DBM sample, there were 4473 (97 %) unique women, and in the TBM sample, 4089 (97 %) were unique women, meaning that less than 3 % of children in the analysis pairs shared the same mother. The distribution of the DBM and TBM samples is displayed in Table 2. Briefly, about a fifth of the children in both samples (DBM 21 %, TBM 23 %) were aged between 12 months and 23 months, and a slight majority were female (DBM 52 %, TBM 52 %). Furthermore, three-quarters of the mothers were aged between 20 and 34 years (DBM 74 %, TBM 74 %), and two-thirds had attended up to primary school education at the time of the survey (DBM 66 %, TBM 66 %).

**Table 2.** Socio-demographic characteristics among mother–child pair analysis samples for DBM (*n* 4618) and TBM (*n* 4209) from the Malawi 2015–2016 Demographic and Health Survey

Variable	Sample for DBM estimation			Sample for TBM estimation		
	Total (*n*)	%	95 % CI	Total (*n*)	%	95 % CI
Age of child						
< 12 months	901	19·51	18·16, 20·93	532	12·65	11·46, 13·93
12–23 months	989	21·41	19·99, 22·90	979	23·27	21·75·24·87
24–35 months	907	19·65	18·29, 21·08	900	21·38	19·90, 22·95
36–47 months	945	20·47	19·08, 21·93	936	22·24	20·72, 23·83
48–59 months	876	18·97	17·68, 20·33	861	20·46	19·09, 21·90
Sex of child						
Male	2234	48·38	46·66, 50·10	2031	48·25	46·52, 49·98
Female	2384	51·62	49·90, 53·34	2178	51·75	50·02, 53·48
Parity						
1 or 2	1902	41·19	39·05, 43·36	1695	40·28	38·05, 42·55
3+	2716	58·81	56·64, 60·95	2514	59·72	57·45, 61·95
Age of mother in years						
15–19	318	6·88	5·98, 7·00	247	5·88	5·06, 6·82
20–34	3427	74·20	72·42, 75·92	3134	74·45	72·60, 76·23
35+	874	18·92	17·39, 20·55	828	19·67	18·04, 21·41
Mother’s marital status						
Not in a union	722	15·63	14·12, 17·27	667	15·85	14·29, 17·54
In a union	3896	84·37	82·73, 85·88	3542	84·15	82·46, 85·71
Highest level of completed education of mother						
None	596	12·91	11·53, 14·43	545	12·94	11·53, 14·50
Primary	3039	65·80	63·77, 67·77	2770	65·80	63·75, 67·78
Secondary and higher	983	21·29	19·56, 23·12	895	21·26	19·50, 23·14
Mother’s employment status						
Not working	1508	32·66	30·46, 34·93	1311	31·14	29·87, 34·50
Working	3110	67·34	65·07, 69·54	2856	67·86	65·50, 70·13
Mother attended < 4 ANC visits						
No	2739	59·31	57·48, 61·11	2526	60·01	58·06, 61·93
Yes	1879	40·69	38·89, 42·52	1683	39·99	38·07, 41·94
Household wealth index						
Low	1573	34·06	32·17, 36·01	1422	33·79	31·83, 35·80
Middle	1527	33·07	31·23, 34·97	1388	32·98	31·06, 34·97
High	1518	32·87	30·83, 34·97	1399	33·23	31·11, 35·42
Area of residence						
Urban	626	13·55	12·36, 14·84	563	13·38	12·09, 14·79
Rural	3992	86·45	85·16, 87·64	3646	86·62	85·21, 87·91
	Mean	sd		Mean	sd	
Community-level variables (mean and sd)						
Proportion of households belonging to the middle, rich or richest wealth quintiles	0·577	0·31	0·557, 0·575	0·565	0·31	0·556, 0·574
Proportion of women with fewer than four ANC visits	0·592	0·23	0·586, 0·600	0·591	0·23	0·584, 0·598

DBM, double burden of malnutrition; TBM, triple burden of malnutrition; N, total sample size across outcome variable; *n*, column sample size across independent variable; ANC, antenatal care; %, percentage.

**Figure 1.**
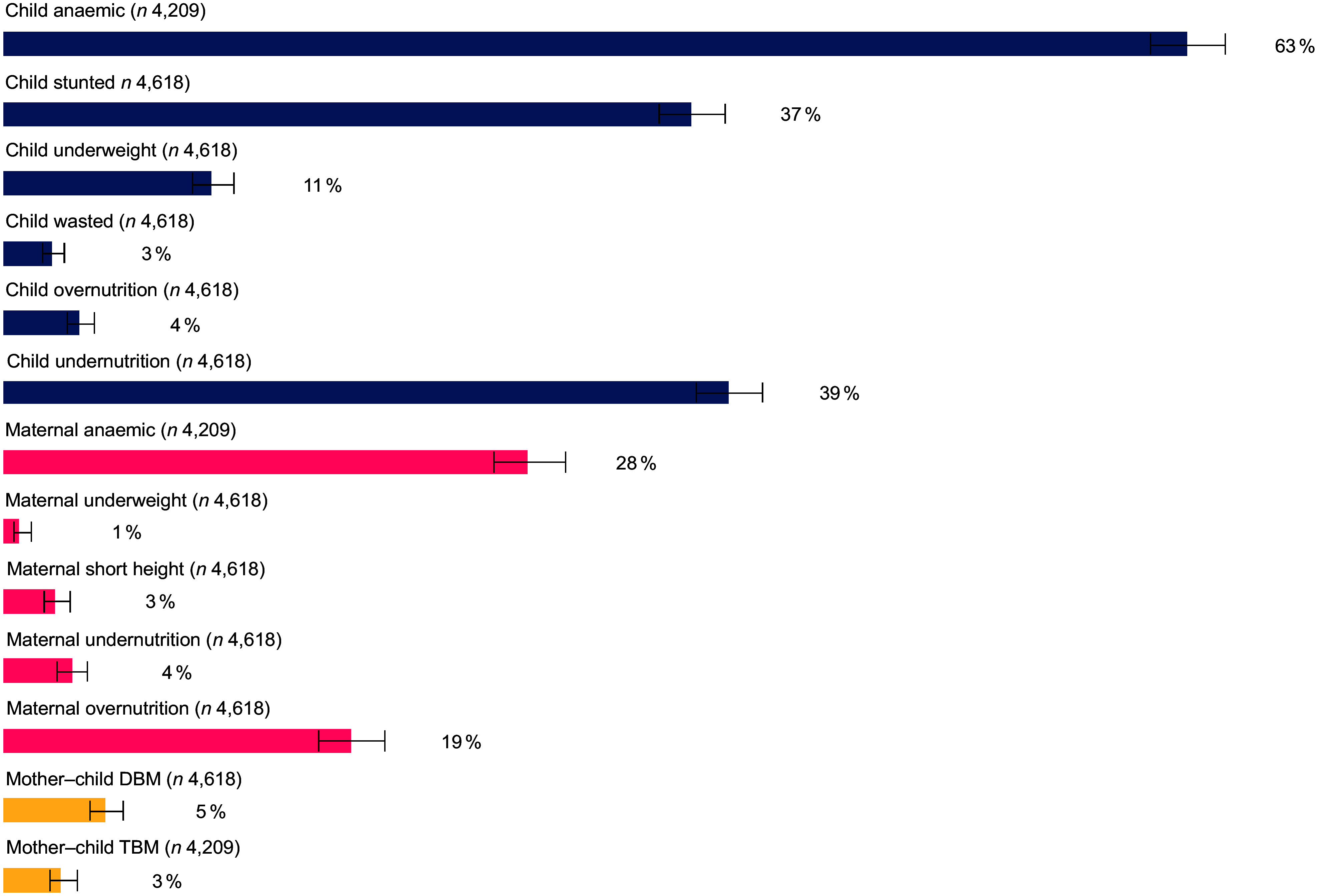
Prevalence of measures of malnutrition among mother–child pairs included in the analysis in Malawi (double burden of malnutrition (DBM): *n* 4618, triple burden of malnutrition (TBM): *n* 4209).

The prevalence of DBM among mother–child pairs in Malawi was 5·5 % (95 % CI: 4·7, 6·4), and the prevalence of TBM was 3·1 % (95 % CI: 2·5 %, 4·0 %). Figure 1 shows the prevalence of the components of DBM and TBM. Anaemia prevalence was 63·4 % among children 6–59 months, and among child undernutrition indicators (child stunted, child underweight and child wasted), stunting was highest at 36·8 %.

### Factors associated with double burden of malnutrition and triple burden of malnutrition

The distribution of characteristics of mother–child pairs according to whether they had DBM or TBM is displayed in Table 3. In bivariate analysis, DBM was associated with older ages of the child and mother, parity and highest level of education of the mother. On the community level, the percentage of households in at least the middle wealth quintile was associated with higher DBM, whilst the proportion of women who attended fewer than four antenatal care (ANC) visits during pregnancy was also associated with lower DBM. In a bivariate analysis of TBM, only parity and maternal education were significantly associated with TBM. Mothers with some education had lower odds of mother–child TBM.

**Table 3.** Bivariate and multivariable analyses (from the multilevel logistic regression model) of the individual, household and community-level variables associated with mother–child pair DBM (*n* 4618) and TBM (*n* 4209), 2015–2016 Malawi Demographic and Health Survey

Variable	DBM sample			DBM model		TBM sample			TBM model	
	*n*	%	*P*-value	AOR	95 % CI	*n*	%	*P*-value	AOR	95 % CI
Individual-level variables										
Age of child			**< 0·001**					0·354		
< 12 months	30	11·2		Ref		15	10·5		Ref	
12–23 months	32	11·7		1·1	0·4, 2·9	24	17·0		0·8	0·3, 2·2
24–35 months	69	25·9		**4·3**	**1·6, 11·8**	40	28·4		1·7	0·6, 4·9
36–47 months	60	22·4		2·1	0·8, 5·2	28	19·6		0·8	0·3, 2·4
48–59 months	77	28·8		**4·1**	**1·4, 11·7**	35	24·5		1·1	0·3, 3·8
Sex of child			0·691					0·670		
Male	125	46·8		Ref		64	45·5		Ref	
Female	143	53·2		0·9	0·5, 1·8	77	54·5		1·2	0·7, 2·1
Parity			**< 0·001**					**0·010**		
1 or 2	72	26·8		Ref		36	25·5		Ref	
3+	196	73·2		1·9	0·9, 4·1	105	74·5		2·0	1·0, 4·2
Age of mother in years			**< 0·001**					0·101		
35+	8	3·0		Ref		6	4·2		Ref	
15–19	174	65·1		0·4	0·1, 2·2	94	66·3		1·1	0·3, 5·1
20–34	86	31·9		**0·4**	**0·2, 0·8**	42	29·5		0·8	0·3, 2·0
Mother’s marital status			0·165					0·570		
Not in a union	32	11·8		Ref		19	13·6		Ref	
In a union	236	88.		0·5	0·2, 1·2	122	86·4		0·7	0·3, 1·6
Highest completed level of education of mother			**< 0·001**					**< 0·001**		
None	47	17·4		Ref		34	24·4		Ref	
Primary	159	59·4		0·6	0·2, 1·3	91	64·8		**0·4**	**0·2, 0·8**
Secondary and higher	62	23·2		0·9	0·3, 2·8	15	10·8		**0·2**	**0·1, 0·6**
Mother’s employment status			0·988					0·967		
Not working	87	32·6		Ref		46	32·4		Ref	
Working	181	67·4		0·8	0·4, 1·9	95	67·6		0·8	0·4, 1·6
Mother attended at least 4 ANC visits			0·876					0·393		
Yes	160	59·9		Ref		79	55·9		Ref	
No	108	40·1		1·1	0·6, 2·0	62	44·1		0·8	0·4, 1·4
Household-level variables										
Household wealth index			0·594					0·388		
Low	81	30·1		Ref		56	39·8		Ref	
Middle	91	34·1		0·9	0·4, 2·0	48	34·0		0·7	0·3, 1·5
High	96	35·8		0·7	0·2, 1·9	37	26·2		0·6	0·2, 1·7
Area of residence			0·414					0·147		
Urban	43	15·9		Ref		12	8·3		Ref	
Rural	225	84·1		1·2	0·6, 2·6	129	91·7		1·5	0·5, 4·4
Community-level variables										
Proportion of households belonging to the middle, rich or richest wealth quintiles	0·63	0·30	**< 0·001**	**2·8**	**1·1, 7·3**	0·58	0·29	0·499	1·5	0·4, 4·8
Proportion of women with fewer than 4 ANC visits	0·56	0·24	**0·032**	0·5	0·2, 1·6	0·58	0·24	0·570	0·4	0·1, 2·1
Random effects										
Cluster variance				33·9	22·7, 50·5				1·4	0·5, 4·3
Household variance				2·1	0·8, 5·6				4·1	1·2, 13·5

DBM, double burden of malnutrition; TBM, triple burden of malnutrition; *n*, column sample size across independent variable (DBM = yes, TBM = yes); *P*-value = Chi-square *P*-value for categorical variables and *t*-test *P*-value for continuous (cluster-level) variables; AOR, adjusted OR; Ref, reference category, AOR, 1; ANC, antenatal care; %, percentage; bold, statistical significance in the multilevel modelling (*P*-value < 0·05).

In the adjusted multilevel logistic regression, an increase in the child’s age was associated with an increase in adjusted odds of DBM. Being a child between age 24 and 35 months is associated with four times higher odds of DBM (adjusted Odds Ratio, AOR: 4·3; 95 % CI: 1·6, 11·8) compared with children aged below 12 months. Similarly, being a child between age 48 and 59 months is associated with four times higher odds of DBM (AOR: 4·1, 95 % CI: 1·4, 11·7) compared with children aged below 12 months. Conversely, the odds of having a mother–child pair with DBM was 60 % lower (AOR: 0·4; 95 % CI: 0·2, 0·8) among mother–child pairs with women aged between 20 and 34 years compared with women aged 35 years and above. In the multilevel logistic regression for TBM among mother–child pairs, the mother’s level of educational attainment was the only factor statistically significantly associated with the outcome (*P*-value < 0·01). The odds of TBM were 60 % lower (AOR: 0·4; 95 % CI: 0·2, 0·8) among mother–child pairs where the women had primary education and 80 % lower (AOR: 0·2; 95 % CI: 0·1, 0·6) among mother–child pairs where the women had secondary or tertiary education, compared with mother–child pairs where the mother had no education.

Sex of the child, parity, mother’s marital status, mother’s employment status, number of attended ANC visits, household wealth quintile and community level of ANC attendance were not associated with DBM or TBM (*P*> 0·05). DBM among mother–child pairs was significantly associated with household wealth in the community. A one-unit increase in the percentage of households in the middle wealth quintile or wealthier in the community was associated with a threefold increase in the adjusted odds of DBM (AOR: 2·8 95 % CI: 1·1, 7·3). No statistically significant community-level effects were seen in the TBM model.

In the spatial analysis, an increase in the nightlight value in an area, which is a proxy for the wealth index, was associated with decreased odds of child-level outcomes such as child stunting, anaemia and underweight. On the other hand, an increase in nightlight value in an area was associated with increased odds of maternal overnutrition (overweight and obese). Likewise, an increase in the proportion of literate women was associated with reduced odds of childhood stunting, wasting, underweight and anaemia. With respect to maternal-level outcomes, an increase in the proportion of literate women was associated with increased odds of maternal overnutrition. Climatic variables such as precipitation, temperature and aridity index were not significantly associated with any of the child-level and maternal-level outcomes.

### Geographic distribution of double burden of malnutrition and triple burden of malnutrition

**Figure 2.**
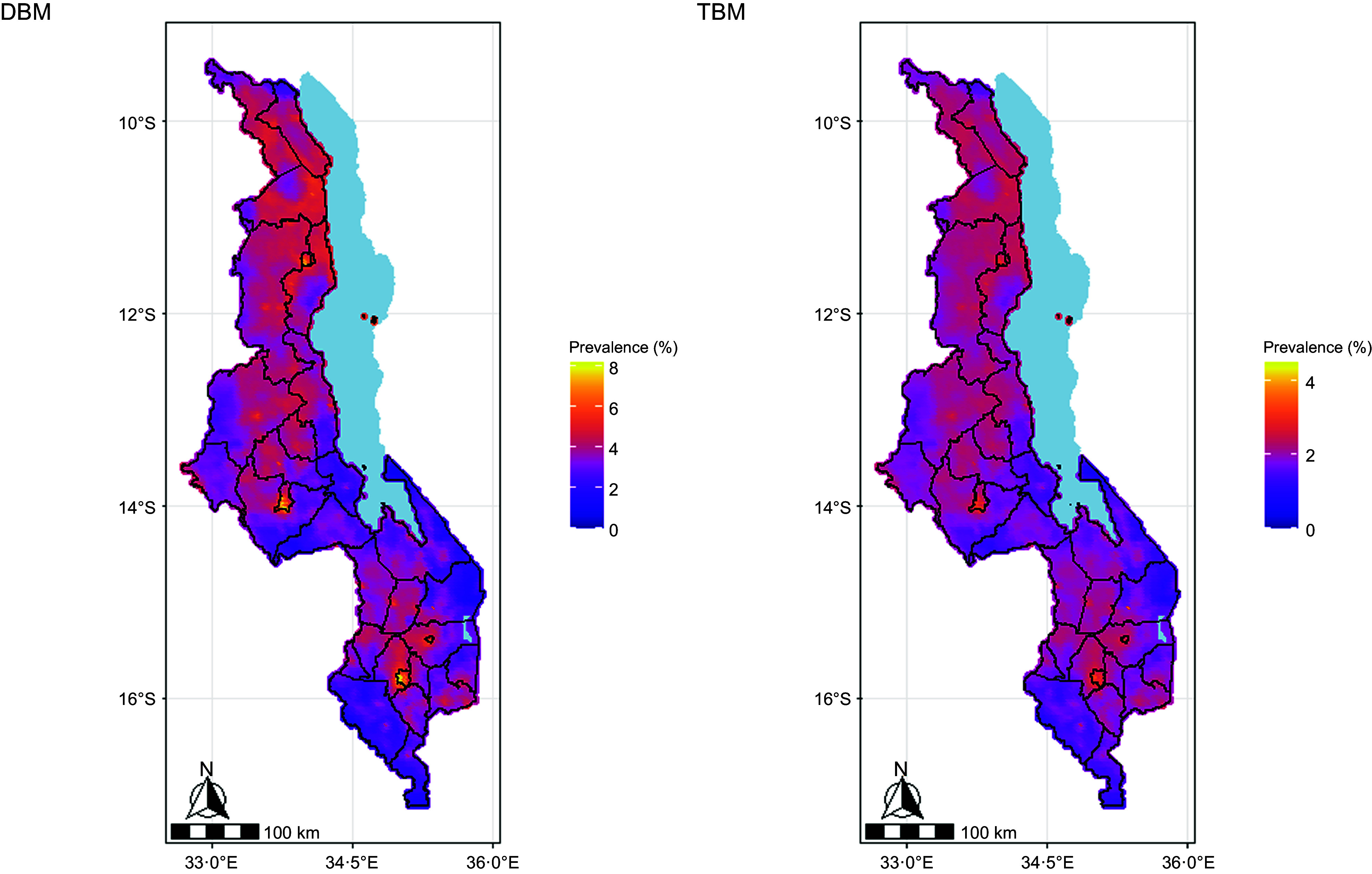
Predicted prevalence of the double burden of malnutrition (DBM) and triple burden of malnutrition (TBM) among mother–child pairs in Malawi.

**Figure 3.**
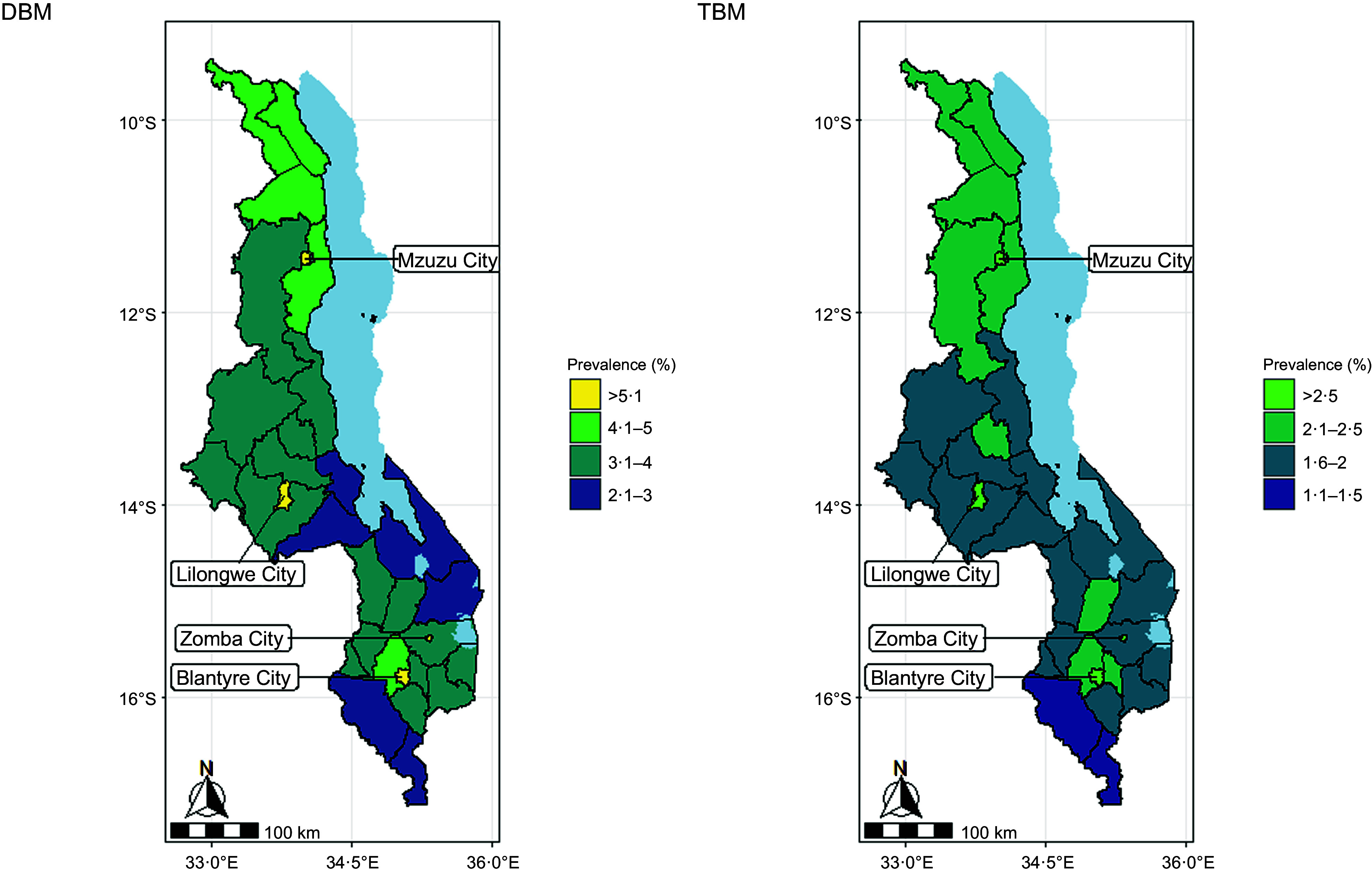
District-level predicted prevalence of the double burden of malnutrition (DBM) and triple burden of malnutrition (TBM) among mother–child pairs in Malawi.

The predicted prevalence of DBM was heterogeneous, ranging from 1·2 % to 8·2 % across the pixels in Malawi (Fig. 2). The predicted prevalence of TBM ranged from 0·9 % to 3·5 % at the pixel level. The highest prevalence of mother–child pairs with DBM and TBM was estimated in cities (Fig. 3). All the four cities in Malawi (Blantyre, Lilongwe, Mzuzu and Zomba) had a DBM prevalence of greater than 5·1 %, and a TBM predicted prevalence greater than 2·4 %. Maps illustrating the uncertainties in the predicted prevalence of DBM and TBM are included in the online supplementary material, Supplemental Information II.

## Discussion and Conclusion

### Discussion

This study examined the prevalence of and spatial variation in DBM and TBM, as well as individual, household and community-level correlates of DBM and TBM among mother–child pairs in Malawi. Our study contributes to the current literature by investigating the multilevel factors associated with DBM and TBM and generating high spatial resolution maps of DBM and TBM in Malawi. The prevalence of DBM and TBM among mother–child pairs were 5·5 % and 3·1 %, respectively. These prevalence values are higher than the reported DBM and TBM prevalence in Ethiopia (3·1 % and 1·6 %, respectively) and similar to the prevalence reported in Nepal (6·6 % and 7·0 %) and India (TBM, 5 %), respectively^(4,15,21)^. Although the overlap between child undernutrition and maternal overweight was small, two-fifths of children were malnourished (39 %), and 19 % of women were overweight or obese. Whilst there was an absence of spatial correlation in our data, our findings suggest that despite the relatively low prevalence, the geographic distribution of mother–child DBM and TBM varied greatly within Malawi, thereby suggesting that environmental conditions conducive for DBM and TBM exist throughout Malawi, particularly in cities. We found a strong positive association between the increased age of a child and the odds of having mother–child pair DBM and TBM. This result is consistent with results from other studies where children older than 24 months had higher odds of being in a mother–child pair with DBM and TBM compared with younger ones^(4,9,15,21,23,40)^. Regarding age, our results also revealed that mother–child pairs where the mother was aged between 20 and 34 had reduced odds of DBM compared with women aged at least 35 years. This finding is consistent with previous studies where the odds of household-level DBM and TBM were higher among older women than younger women due to the older women being overweight and obese^(9,41)^. Some studies have postulated that sedentary lifestyles and the increased likelihood of overnutrition (overweight and obesity) among older women might be some of the leading drivers of DBM and TBM^(4,9,15)^.

Educational attainment was another predictor of TBM in our study. Mothers with at least primary and secondary education had reduced odds of TBM. This result further corresponds with the spatial modelling where an increase in the proportion of literate women was associated with a reduced burden of childhood malnutrition (underweight, stunting and wasting). These results align with previous evidence that women with higher education have reduced odds of household-level DBM and TBM as higher education attainment is associated with reduced risk of childhood malnutrition^(9,23)^.

Our results show that an increase in the proportion of wealthy households at the community level increased the odds of DBM, thereby suggesting a higher burden in relatively wealthier areas^(8,21)^. The spatial analysis further decomposes this finding by showing that an increase in the nightlights value (a proxy for the level of wealth) is associated with reduced odds of child-level outcomes and increased odds of maternal overnutrition. No significant community-level effects were seen in the TBM model. Previous work has postulated that higher income levels may enhance the ability to buy food, thereby shaping dietary habits and preferences^(41)^. Furthermore, previous studies have hypothesised that inadequate physical activity, sedentary lifestyles and westernised dietary behaviours adopted by wealthier households and communities drive adult overnutrition^(8,21,41,42)^. Previous reports have shown that these behaviours tend to be more common in urban settings, which could be reflected in our results^(43)^. Further research is needed in Malawi to identify more community-level determinants of DBM and TBM.

The absence of spatial correlation in our data suggests that the spatial correlation in our DBM and TBM samples can be explained by the environmental covariates that were used in the models. This notwithstanding, the maps generated in this study suggest that DBM and TBM are not homogeneously distributed in Malawi. These results underscore the potential existence of spatial drivers influencing the observed patterns of DBM and TBM, which should be further investigated. In studies where the data exhibit spatial correlation, the mixed effects models used in this study could be further extended to explicitly incorporate the spatial correlation using geostatistical methods^(36,37)^.

### Limitations and strengths of the study

The main limitation of this study is that the DHS was a cross-sectional study; therefore, no causal associations could be inferred from the study. Second, our analysis included categorising continuous variables such as wasting and stunting that have been shown to lead to some loss of information on the variable^(44)^. An additional constraint pertains to the use of outdated DHS data. Nevertheless, this research remains valuable as it demonstrates the application of mixed effects models for estimating factors at multiple levels and mapping health outcomes like DBM and TBM. Another limitation of DHS data is the unavailability of most of the variables in the WHO DBM conceptual framework. Furthermore, the DHS data lack information on dietary behaviours that are immediately related to nutritional outcomes. This shortcoming creates the need to have more nutrition-related datasets in Malawi to better understand the determinants of nutrition-related issues such as DBM and TBM in the country. Another important limitation is the lack of micronutrient data. Thus, we used anaemia status as a proxy for estimating TBM prevalence. We, therefore, recommend collecting nationally representative data on micronutrient deficiencies and not necessarily through DHS.

Given that common household-level risk factors for child undernutrition are often protective for maternal overweight and vice versa, most of the risk factors identified for DBM and TBM in this study were driven either by strong positive associations with child undernutrition (and weaker or null negative associations with maternal overnutrition) or by strong positive associations with maternal overnutrition (and weaker or null negative associations with child undernutrition). These limitations might explain why the prevalence of DBM and TBM appears possibly lower than what would be observed by chance. These methodological challenges are, nevertheless, common when studying DBM and TBM.

Despite these limitations, to our knowledge, this study is the first to establish the prevalence of mother–child pair DBM and TBM in Malawi. Our study also presents an important finding that the coexistence of undernutrition in children and overnutrition in mothers is associated with both individual and community-level factors. Furthermore, our study is the first to illustrate the geographical disparities in the distribution of mother–child DBM and TBM in Malawi. It shows that the burden of DBM and TBM is higher in cities than in other areas. Future studies could further extend this work by building a spatiotemporal model to assess whether the burden of DBM and TBM has also been higher in cities than in other areas over time. This is because a recent study has shown a shifting spatiotemporal trend in DBM from metropolitan areas to other regions in Guatemala^(45)^. The findings from the spatiotemporal modelling could help the government and implementing partners anticipate which districts/areas in Malawi might get an increasing burden of DBM and TBM in the future. The study results could also inform interventions about specific areas where attention is needed to target DBM and TBM and control the burden of these conditions. The findings from this study could also be used to inform a new hypothesis of shifting spatial DBM and TBM trends in other countries.

### Conclusion

This study highlighted that the mother–child pair prevalence of DBM and TBM in Malawi was relatively low, with the highest burden in cities. It further showed that the increased age of the child and mother also increased the odds of a mother–child pair having DBM. Additionally, individual-level maternal educational attainment was shown to have a protective effect against TBM. Our study also highlighted that there are community-level determinants of DBM such as household wealth that are associated with increased odds of DBM. These results emphasise the need to not neglect wealthier communities in disseminating and implementing adult-related nutrition-related interventions in Malawi. These results could be explicitly adopted and translated into national documents such as the ‘Malawi National Multi-Sector Nutrition Policy 2018–2022’, which acknowledges the existence of both undernutrition and overnutrition but does not provide any recommendations on addressing their coexistence such as DBM and TBM^(46)^.

Through the revised Malawi National Multi-Sector Nutrition Policy, the government of Malawi can tackle DBM and TBM among mother–child pairs in Malawi by implementing comprehensive, integrated nutrition programmes that simultaneously address undernutrition in children and overnutrition in mothers. The government can also promote a multisectoral response to dealing with mother–child DBM and TBM in Malawi. A multisectoral approach can ensure that policies across multiple sectors, such as health and agriculture, work together to ensure access to supplements and nutritious foods, address food insecurity and promote healthy habits among women. These interventions could, as a priority, begin to focus on the Malawian cities where DBM and TBM among mother–child pairs are the highest.

## Supporting information

Khaki et al. supplementary material 1 Khaki et al. supplementary material

Khaki et al. supplementary material 2 Khaki et al. supplementary material

## Supplementary material

For supplementary material accompanying this paper, visit https://doi.org/10.1017/S1368980024002064.
