# Supplementary material for: Prevalence and determinants of double and triple burden of malnutrition among mother–child pairs in Malawi: a mapping and multilevel modelling study
Source: Public Health Nutr. 2024 Oct 21;27(1):e241. doi: 10.1017/S1368980024002064 (PMC11645115; doi:10.1017/S1368980024002064)
Supplement: Khaki et al. supplementary material 1 — Khaki et al. supplementary material [file S1368980024002064sup001.docx]

**Supplementary information II for “Prevalence and determinants of double and triple burden of malnutrition among mother-child pairs in Malawi: a mapping and multilevel modelling study”**

**Contents**

[2.1 Sources of spatial covariates 2](#_Toc169767594)

[2.2 WHO Conceptual Framework for the Double Burden of Malnutrition 5](#_Toc169767595)

[2.3 Definitions of variables used in the analysis 6](#_Toc169767596)

[2.4 Multilevel binomial mixed effects model 7](#_Toc169767597)

[2.5 Exploratory analysis for spatial correlation using the theoretical variogram 7](#_Toc169767598)

[2.6 Model estimates 10](#_Toc169767599)

[2.7 Additional results 12](#_Toc169767600)

[2.8 Model predictions 14](#_Toc169767601)

# **Sources of spatial covariates**

A gridded continuous map of the 2015 nightlight data for Malawi was downloaded from the Worldpop website^(1)^. The Worldpop is a research centre that models and develops gridded continuous maps, otherwise known as raster datasets, to aid in mapping and geospatial modelling. The elevation raster for Malawi was also downloaded from Worldpop^(1)^. Both the nightlight and elevation rasters had spatial resolutions of 100$m^{2}$.

Climate data, including temperature, precipitation and evapotranspiration were extracted from the TerraClimate website for 2015^(2)^. We computed an aridity index by taking the proportion of the precipitation to the evapotranspiration. The climate data are characterized by a monthly time interval and a spatial resolution of approximately 4 km (equivalent to 1/24th of a degree).

We also used spatial covariates generated by the Demographic and Health Survey (DHS) Program. We, specifically, obtained data on the percentage of women aged 15−49 who are literate, percentage of children 12−23 months who had received all 8 basic vaccinations and percentage of women who had a live birth in the five years preceding the survey who had 4 or more antenatal care visits^(3–5)^. All the DHS rasters were modelled using the 2015-16 Malawi DHS and had a resolution of 5km x 5km.


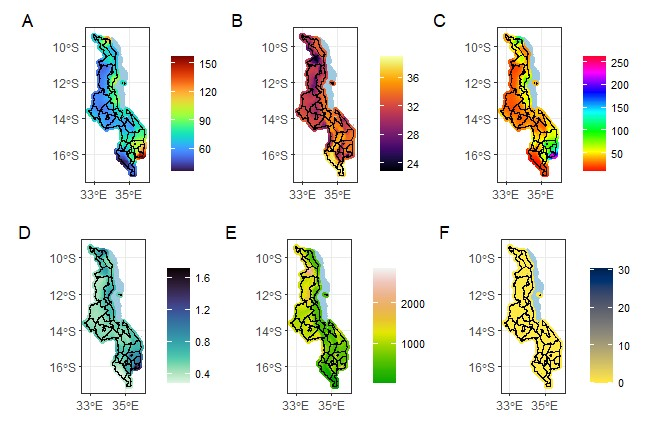


Figure S1: Maps of spatial covariates used to model and map the prevalence of DBM and TBM.

A = Precipitation (mm); B = Maximum temperature (^0^C); C = Evapotranspiration (mm); D= Aridity index (ratio of Precipitation to Evapotranspiration); E = Elevation (m); F = Nightlight (nanoWatts/cm2/sr).


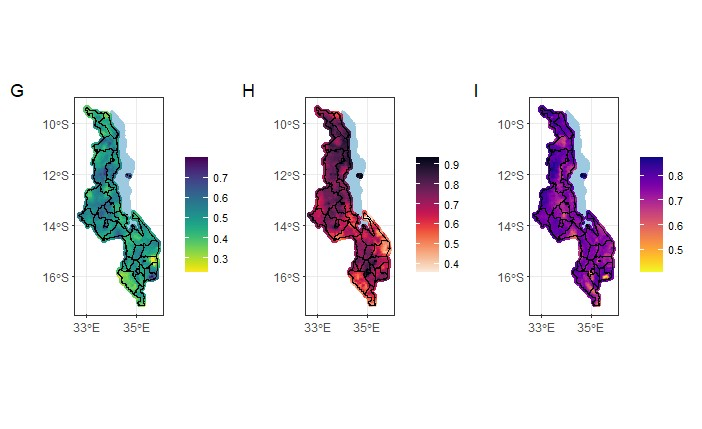


Figure S2: Maps of spatial covariates used to model and map the prevalence of DBM and TBM. G = Percentage of women who had a live birth in the five years preceding the survey who had 4+ antenatal care visits; H = Percentage of women aged 15−49 who are literate; I = Percentage of children 12−23 months who had received all 8 basic vaccinations.

# **WHO Conceptual Framework for the Double Burden of Malnutrition**


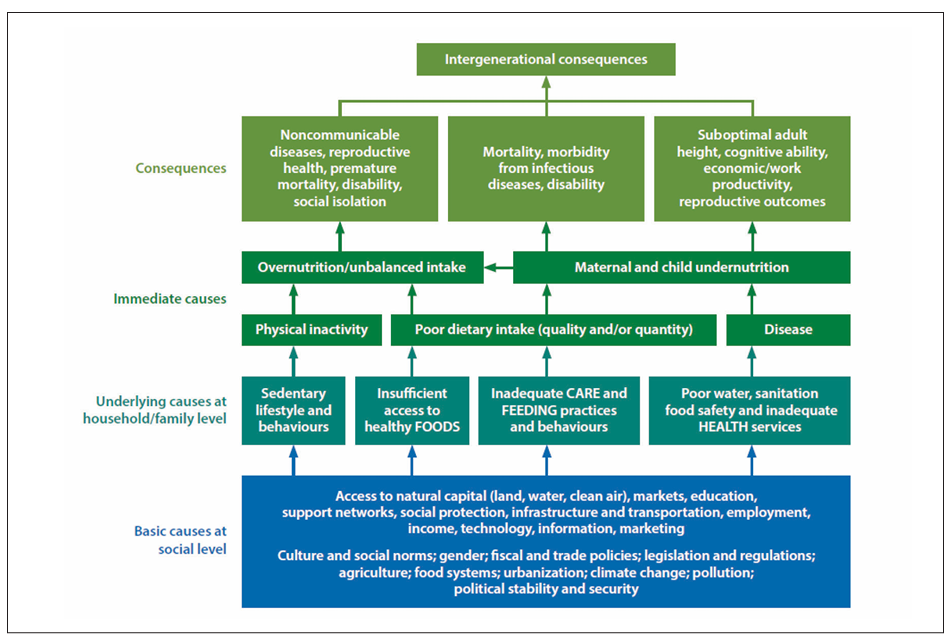


Figure S3: The World Health Organization Conceptual Framework for DBM

# **Definitions of variables used in the analysis**

Table S1: Definitions of variables used in the analysis

| **Variable name** | **Definition** | **Variable code(s)** |
| --- | --- | --- |
| **Mother-child pair variables** |  |  |
| Age group of child in months | <12, 12-23, 24-35, 36-47, 48-59 months | B8 |
| Sex of child | Male, Female | B4 |
| Age group of mother at the time of the survey | 15-19, 20-34, 35+ years | V013 |
| Mother’s highest completed education level | None, Primary (incomplete and complete), secondary (incomplete and complete), and higher | V149 |
| Mother’s marital status at the time of the survey | In a union (married or cohabiting) and not in a union (never been in a union, single, divorced, or widowed) | V502 |
| Mother’s employment status | Not working, working | V714 |
| Parity | <2, 2+ | V201 |
| Fewer than 4 ANC visits during pregnancy with most recently born child | Yes (<4 ANC visits), No (≥ 4 visits) | M14 |
|  |  |  |
| **Household-level variables** |  |  |
| Household wealth index | Low, middle, high | V191 |
|  |  |  |
| **Community-level variables** |  |  |
| Area of residence | Urban, Rural | V025 |
| Proportion of households in middle or rich wealth quintiles in a cluster | Total number of households with middle or higher wealth quintile in a cluster, divided by total number of households in a cluster | V190, V001 |
| Proportion of women with fewer than 4 ANC visits during pregnancy of child in mother-child pair in a cluster | Total number of mothers who attended less than 4 ANC visits in a cluster, divided by the total number of mothers in that cluster | V190, V001 |

# **Multilevel binomial mixed effects model**

Let $Y_{jk}$be the random variable denoting the number of mother-child pairs with DBM or TBM out of the total number of mother-child pairs in a household $j$ that is nested within a cluster $k$. We then modelled the outcome variable $Y_{jk}$ using a three-level binomial mixed effects model with probability ($p(x_{jk})$) of having DBM or TBM such that:

$$\log\left\{ \frac{p(x_{jk})}{1-p(x_{jk})} \right\} = d\left( x \right)^{T}\beta+ \psi_{k}+ \delta_{jk}$$

Where $d(x)$ is a vector of variables associated with the regression coefficients β, and $\psi_{k}$ is the effect of cluster/community $k (k=1,2,\ldots,K)$ and $\delta_{jk}$ is the effect of household $j (j=1,2,\ldots,J)$ within cluster $k$. The random effects ($\psi_{k}+ \delta_{jk}$) are assumed to be independent of one another and are normally distributed with zero means and constant variances {($\psi_{k} \sim N\left( 0, \sigma_{k} \right), \delta_{jk}\sim N(0,\sigma_{jk})$}^(6,7)^. The individual, household, and cluster-level independent variables that were included in the analysis of DBM and TBM are described in the main manuscript.

# **Exploratory analysis for spatial correlation using the theoretical variogram**

To assess whether there was any spatial correlation in DBM and TBM in Malawi, we fitted the binomial mixed model defined above and we included the georeferenced covariates described in section 2.1 above. We fitted this model to all the 9 outcomes (child wasting, child stunting, child underweight, child overweight, child anaemia, maternal short height, maternal underweight, maternal overnutrition, and maternal anaemia) in R using the lme4 package. To test for spatial correlation in the data, we extracted the random effects ($\psi_{k}+ \delta_{jk}$) from the mixed effects model for each of the 9 models and fitted them to a variogram^(8–10)^. We then generated 95% confidence intervals of the random effects variograms under the assumption of spatial independence. We concluded that there was no evidence of spatial correlation in the data since the variograms for all the 9 models fell within the 95% confidence bounds (Figure S4 and Figure S5). We, therefore, fitted non-spatial mixed effects models for all the 9 outcomes and not geostatistical models since the data did not show any evidence of residual spatial correlation^(10,11)^. These models were used to compute continuous predictions of the 9 outcomes at 3 Km^2^ grids and at district level.


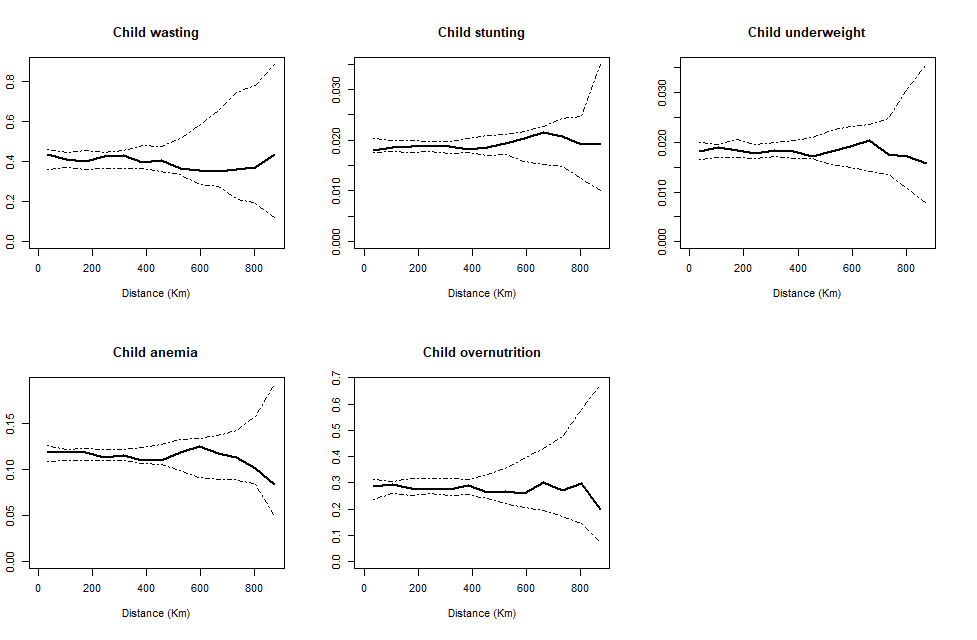


Figure S4: Plot of the empirical variogram (represented by the black solid line) based on the random effects from Binomial mixed models for the child-level outcomes. The dotted lines correspond to 95% confidence intervals generated under the assumption of spatial independence ^11^.


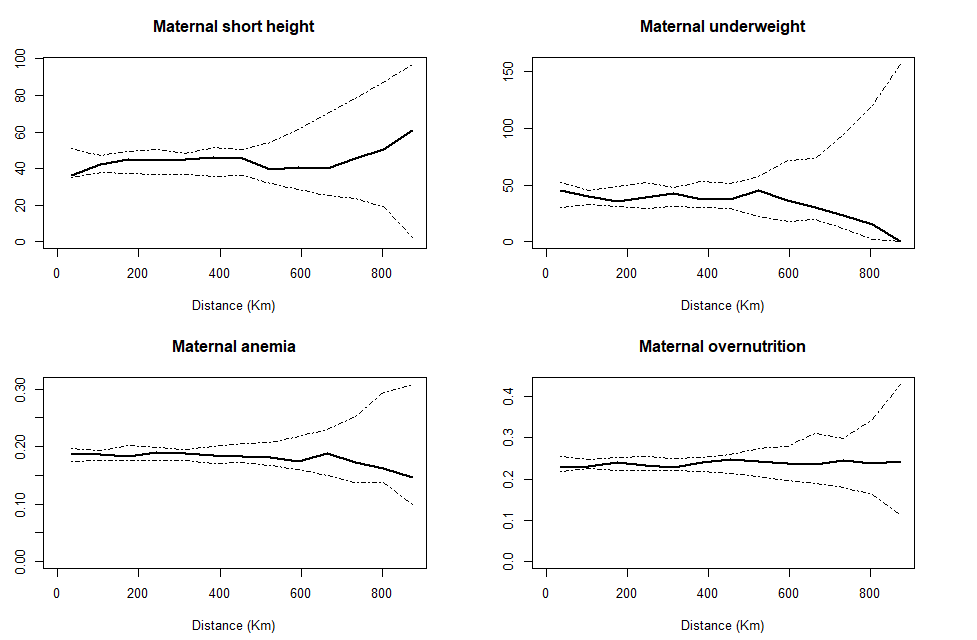


Figure S5: Plot of the empirical variogram (represented by the black solid line) based on the random effects from Binomial mixed models for the maternal-level outcomes The dotted lines correspond to 95% confidence intervals generated under assumption of spatial independence ^11^.

# **Model estimates**

Table S2: Model parameter estimates and associated 95% confidence intervals (CI) for child-level outcomes

| **Variable** | **Child underweight** | **Child wasting** | **Child stunting** | **Child anaemia** | **Child overnutrition** |
| --- | --- | --- | --- | --- | --- |
|  | **Estimate (95% CI)** | **Estimate (95% CI)** | **Estimate (95% CI)** | **Estimate (95% CI)** | **Estimate (95% CI)** |
| Intercept | -1.26 | -6.87 | -0.01 | 2.17 | -6.48 |
|  | (-2.03, -0.49) | (-11.52, -2.22) | (-0.54, 0.53) | (1.50, 2.93) | (-9.64, -3.31) |
| Nightlight | -0.06 | NA | -0.05 | -0.02 | NA |
|  | (-0.12, 0.001) |  | (-0.09, -0.01) | (-0.06, 0.04) |  |
| Maximum | NA | 0.08 | NA | NA | NA |
| temperature |  | (-0.03, 0.18) |  |  |  |
| Percentage of | -1.14 | -0.39 | -0.79 | -0.21 | -0.61 |
| literate women | (-1.14, -0.08) | (-2.92, 2.14) | (-1.53, -0.05) | (-3.03, -1.06) | (-2.56, 1.33) |
| Proportion of children | NA | NA | NA | NA | 3.90 |
| fully vaccinated |  |  |  |  | (006, 7.74) |

Note: NA corresponds to a situation where the spatial covariate is not included in the model.

Table S3: Model parameter estimates and associated 95% confidence intervals (CI) for maternal-level outcomes

| **Variable** | **Maternal underweight** | **Maternal short height** | **Maternal anaemia** | **Maternal overnutrition** |
| --- | --- | --- | --- | --- |
|  | **Estimate (95% CI)** | **Estimate (95% CI)** | **Estimate (95% CI)** | **Estimate (95% CI)** |
| Intercept | -6.88 | -4.08 | -4.68 | -4.55 |
|  | (-10.43, -3.32) | (-5.60, -2.57) | (-5.90, -3.46) | (-5.45, -3.65) |
| Nightlight | 0.01 | -0.05 | NA | 0.08 |
|  | (-0.13, 0.17) | (-0.16, 0.05) |  | (0.04, 0.12) |
| Maximum | NA | NA | NA | NA |
| temperature |  |  |  |  |
| Percentage of | -0.33 | 0.26 | 0.11 | 3.74 |
| literate women | (-3.90, -3.25) | (-1.80, 2.32) | (0.07, 0.15) | (2.54, 4.94) |

Note: NA corresponds to a situation where the spatial covariate is not included in the model.

# **Additional results**


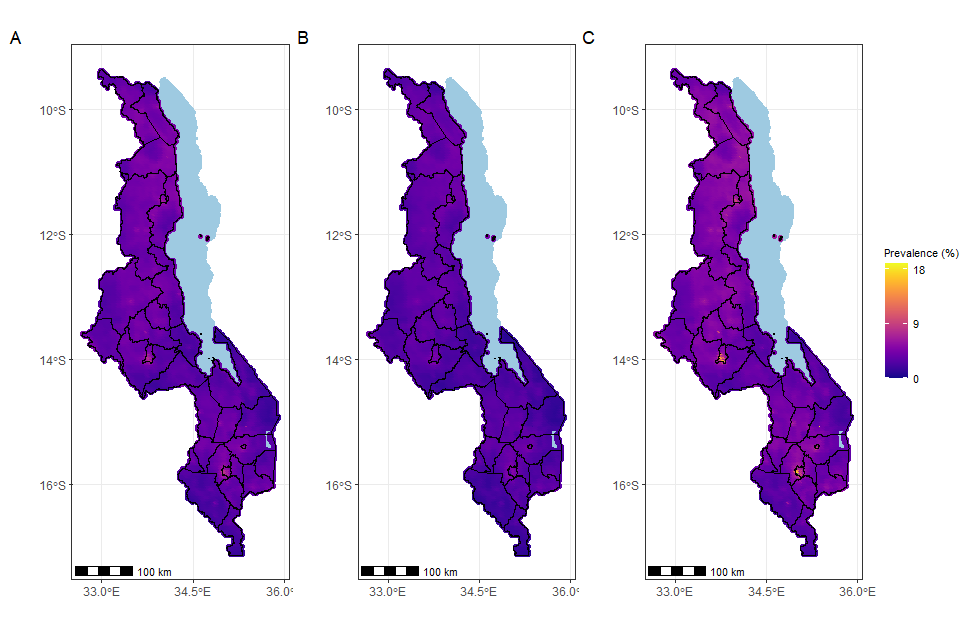


Figure S4: Predicted mother-child pair DBM prevalence maps of Malawi; mean predicted prevalence (A) and, lower (B) and upper 95% CI bounds (C).


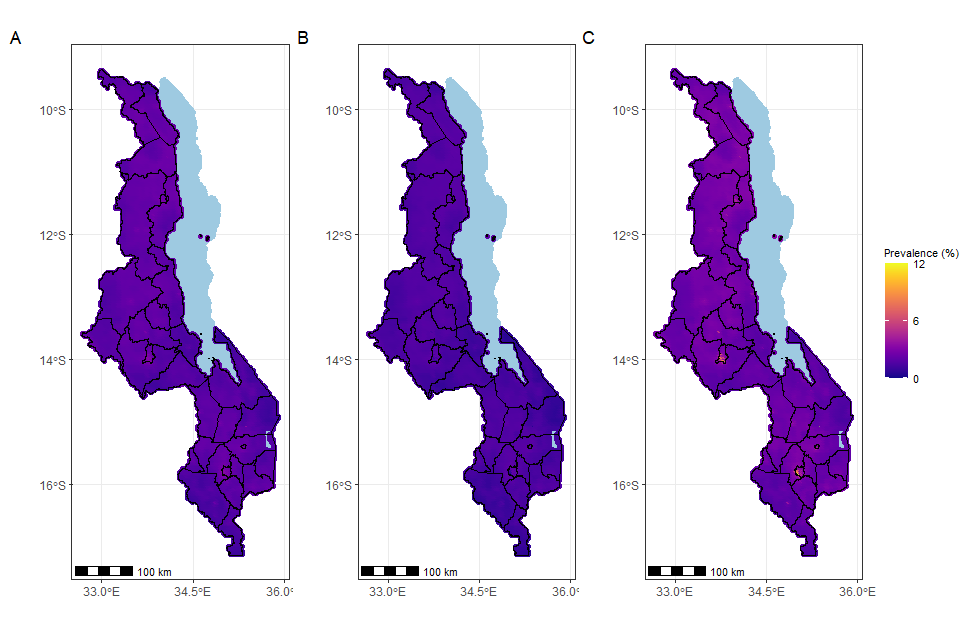


Figure S5: Predicted mother-child pair TBM prevalence maps of Malawi; mean predicted prevalence (A) and, lower (B) and upper 95% CI bounds (C).

# **Model predictions**

We predicted the prevalence of DBM and TBM across Malawi at point-level. We defined our predictive target as $T^{*}$. We divided the continuous surface of Malawi into a regular grid of $3{km}^{2}$. The following equation was used to generate prevalence surfaces for DBM and TBM:

$$T^{*}= \left\{ p\left( x \right):x \in A \right\}$$

Where $T= d\left( x \right)^{T}\beta+ \psi_{k}+ \delta_{jk}$ for each of the 9 outcomes and A denotes the regular grid for Malawi for the point estimates ^(11)^.

To generate maps for DBM and TBM from the non-spatial mixed effects models, we used statistical probability rules that are also employed in other health research that uses outcomes that are derived from multiple indicators^(12)^ as follows. Let:

- P(A) be the probability that a child is stunted
- P(B) probability that a child is wasted
- P(C) probability that a child is underweight
- P(D) be the probability that a child is overweight or obese
- P(E) be the probability that a child is anaemic
- P(F) be the probability that a mother has a short height
- P(G) be the probability that a mother is underweight
- P(H) be the probability that a mother is overweight or obese
- P(I) be the probability that a mother is anaemic.

Then the probability that a child has undernutrition (is stunted or wasted or underweight) is defined as

$$P \left( child undernutrition \right)=1-\left\{ \left( 1-P\left( A \right) \right)\times\left( 1-P\left( B \right) \right)\times(1-P\left( C \right)) \right\}$$

And the probability that a mother has undernutrition (short height or underweight) is defined as:

$$P\left( maternal undernutrition \right)=1- \left\{ \left( 1-P\left( F \right) \right)\times(1-P\left( G \right)) \right\}$$

Consequently, the probability that a mother-child pair has the double burden of malnutrition (DBM1 = child undernutrition and maternal overnutrition OR DBM2 = child overnutrition and maternal undernutrition) is defined as:

$$P \left( DBM1 \right)=P\left( child undernutrition \right)\times P(maternal overnutrition)$$

$$P \left( DBM2 \right)=P\left( child overnutrition \right)\times P(maternal undernutrition)$$

Then the overall double burden of malnutrition (child undernutrition and maternal overnutrition OR child overnutrition and maternal undernutrition) is given as:

$$P\left( Any DBM \right)=1- \left\{ \left( 1-P\left( DBM1 \right) \right)\times(1-P\left( DBM2 \right)) \right\}$$

The probability that a mother-child pair has the triple burden of malnutrition (TBM1 = child undernutrition, maternal overnutrition and child anaemia OR TBM2 = child overnutrition, maternal undernutrition and maternal anaemia) is calculated as follows:

$$P\left( TBM1 \right)=P\left( child undernutrition \right)\times P\left( maternal overnutrition \right)\times P(child anaemia)$$

$$P\left( TBM2 \right)=P\left( child overnutrition \right)\times P\left( maternal undernutrition \right)\times P(maternal anaemia)$$

Similar to the overall double burden of malnutrition, the overall triple burden of malnutrition (child undernutrition, child anaemia and maternal overnutrition OR child overnutrition, maternal undernutrition, and maternal anaemia) is given as:

$$P\left( Any TBM \right)=1- \left\{ \left( 1-P\left( TBM1 \right) \right)\times(1-P\left( TBM2 \right)) \right\}$$

**References**

1. Tatem AJ. WorldPop, open data for spatial demography. *Sci Data*. 2017;4(1):170004. doi:10.1038/sdata.2017.4

2. Abatzoglou JT, Dobrowski SZ, Parks SA et al. TerraClimate, a high-resolution global dataset of monthly climate and climatic water balance from 1958–2015. *Sci Data*. 2018;5(1):170191. doi:10.1038/sdata.2017.191

3. ICF International. Spatial Data Repository, The Demographic and Health Surveys Program. Modeled Surfaces. MW2015DHS_CHVACCCBAS_MS_v01 ICF International. Funded by the United States Agency for International Development (USAID). https://spatialdata.dhsprogram.com/modeled-surfaces/#survey=MW|2015|DHS

4. ICF International. Spatial Data Repository, The Demographic and Health Surveys Program. Modeled Surfaces. MW2015DHS_RHANCNWN4P_MS_v01. https://spatialdata.dhsprogram.com/modeled-surfaces/#survey=MW|2015|DHS

5. ICF International. Spatial Data Repository, The Demographic and Health Surveys Program. Modeled Surfaces. MW2015DHS_EDLITRWLIT_MS_v01.

6. Sommet N, Morselli D. Keep Calm and Learn Multilevel Logistic Modeling: A Simplified Three-Step Procedure Using Stata, R, Mplus, and SPSS. *Int Rev Soc Psychol*. 2017;30(1):203-218. doi:10.5334/irsp.90

7. Leckie, G. Three-Level Multilevel Models - Concepts. LEMMA VLE Module 11, 1-47. Published 2013. https://www.bristol.ac.uk/media-library/sites/cmm/migrated/documents/11-concepts-example.pdf

8. Bates D, Mächler M, Bolker B et al. Fitting Linear Mixed-Effects Models Using **lme4**. *J Stat Softw*. 2015;67(1). doi:10.18637/jss.v067.i01

9. Deribe K, Mbituyumuremyi A, Cano J, et al. Geographical distribution and prevalence of podoconiosis in Rwanda: a cross-sectional country-wide survey. *Lancet Glob Health*. 2019;7(5):e671-e680. doi:10.1016/S2214-109X(19)30072-5

10. Giorgi E, Fronterrè C, Macharia PM et al. Model building and assessment of the impact of covariates for disease prevalence mapping in low-resource settings: to explain and to predict. *J R Soc Interface*. 2021;18(179):20210104. doi:10.1098/rsif.2021.0104

11. Diggle PJ, Giorgi E. *Model-Based Geostatistics for Global Public Health: Methods and Applications*. CRC Press; 2019.

12. De Silva N, Hall A. Using the Prevalence of Individual Species of Intestinal Nematode Worms to Estimate the Combined Prevalence of Any Species. Gyorkos TW, ed. *PLoS Negl Trop Dis*. 2010;4(4):e655. doi:10.1371/journal.pntd.0000655
