# Supplementary material for: Prevalence and determinants of double and triple burden of malnutrition among mother–child pairs in Malawi: a mapping and multilevel modelling study
Source: Public Health Nutr. 2024 Oct 21;27(1):e241. doi: 10.1017/S1368980024002064 (PMC11645115; doi:10.1017/S1368980024002064)
Supplement: Khaki et al. supplementary material 2 — Khaki et al. supplementary material [file S1368980024002064sup002.docx]

Total number of women interviewed in the 2015-16 MDHS (N= 24,562)

**Exclusion Criteria**

- Women who have never had children (n=5,574).

Total number of women with children (n= 18,988)

- Women with no child in 5 years prior to MDHS (n=2,037).
- Total number of women with under-five child in 5 years prior to MDHS (n=16,951)
- Total number of mother-child pairs (16,618 singleton and 333 multiple births; n = 17,286)
- Mother-child pairs whose child died (n=824)
- Mother-child pairs where mother was pregnant at time of survey (n=1,022)
- Mother-child pairs from households that were not selected for hemoglobin and anthropometry tests (n=10,192)
- Mother-child pairs with missing BMI measurements for women (n=97)
- Mother-child pairs with missing height measurements for women (n=4)
- Mother-child pairs with flagged measurements on women or women pregnant in the 2 months prior to survey (n=175)
- Mother-child pairs with missing or flagged measurements on child (n=354)

Total number of mother-child pairs with non-pregnant mother and child who is alive (n=15,440)

Mother-child pairs with complete information on child undernutrition, child overnutrition, mother’s height, and mother’s BMI (N=4,618 **DBM sample**)

- Mother-child pairs where the child did not have their hemoglobin (HB) measurements taken (n=390)
- Mother-child pairs where the mother did not have their HB measurements taken (n=19)

Non-pregnant mother-child pairs with complete information on child undernutrition, child overnutrition, child anemia, mother’s height, mother’s anemia, and mother’s BMI (N=4,209, **TBM sample**)

Figure S1: Flowchart of the sample included in the analysis from the 2015-16 Malawi Demographic and Health Survey (MDHS) (numbers are not weighted)
